# COVID-19 and the emergence of inpatient tele-ward rounds

**DOI:** 10.1192/j.eurpsy.2022.451

**Published:** 2022-09-01

**Authors:** X. Boland, L. Dratcu

**Affiliations:** South London and Maudsley NHS Mental Health Trust, General Adult Psychiatry, London, United Kingdom

**Keywords:** Multidisciplinary team work, Ward rounds, telepsychiatry, Inpatient psychiatry

## Abstract

**Introduction:**

Telemedicine has been at the heart of healthcare system’s strategic response to the COVID-19 pandemic. Within psychiatry, there has been a surge of research and guidelines into the use of video-teleconferencing to replace face to face consultations across clinical settings. Clinical ward rounds are central to inpatient psychiatric care yet little guidance is available on how best to integrate telemedicine into the multidisciplinary work of inpatient psychiatry.

**Objectives:**

We report on the introduction of video teleconferencing for psychiatric ward rounds on our acute inner-London psychiatric unit during the outbreak of COVID-19.

**Methods:**

In undertaking the rapid transition to tele-ward rounds, we had to reconcile the multiple functions of psychiatric ward rounds with the technological resources available to us.

**Results:**

Tele-ward rounds helped simplify care delivery, facilitate multidisciplinary collaboration and improve accessibility for patients and relatives in a time of crisis. The transition to tele-ward rounds also brought about technical, operational and communication issues that may impact on the patient experience and quality of care including governance challenges, contextual dissonance and technological limitations.

**Conclusions:**

The routine use of newer technology in psychiatry ward rounds is unlikely to succeed on the basis of improvisation, particularly given the stream of technical innovations in telemedicine, and the multifarious quality of social interactions in our clinical setting. Staff training and the development of an adapted etiquette and code of communication are both essential. Patient participation in future developments will also help ensure tele-ward rounds continue to meet the standards of high quality inpatient psychiatric care beyond the COVID-19 pandemic.

**Disclosure:**

No significant relationships.

